# Asymmetric breast dose in coronary angiography

**DOI:** 10.1120/jacmp.v17i2.5746

**Published:** 2016-03-08

**Authors:** Kam Shan Mong, Anthony B. Wallace, Rick D. Franich

**Affiliations:** ^1^ Medical Physics Department Austin Health Heidelberg VIC Australia; ^2^ Medical Imaging Section Australian Radiation Protection and Nuclear Safety Agency Yallambie VIC Australia; ^3^ School of Applied Sciences RMIT University Melbourne VIC Australia

**Keywords:** Gafchromic XR‐QA2, coronary angiogram, breast dose, asymmetry

## Abstract

The purpose of this study was to demonstrate asymmetric radiation dose distribution to the breasts in coronary angiography. Gafchromic XR‐QA2 film was used as an area dosimeter to capture the asymmetric dose distribution to the breasts at various tissue depths in an anthropomorphic phantom. A selection of tube angulations were used under a controlled experiment and during a mock coronary angiography procedure. The Gafchromic XR‐QA2 film was able to confirm the asymmetric distribution of radiation dose to the breast and provide a normalized breast dose value. The right breast received the majority of dose for most of the tube angulations in the controlled experiment. However the left breast received the most radiation dose during the mock procedure. Asymmetric dose distribution to the breasts is normally not observed if Monte Carlo based simulations are performed because individual breast dose calculations are not available. The application of a typical coronary angiogram determined in the experiment showed the normalized left breast dose is $0.16\;\text{mGy}/\text{Gy}.{\text{cm}}^{2}$ and the right breast dose is $0.08\;\text{mGy}/\text{Gy}.{\text{cm}}^{2}$.

PACS number(s): 87.53. Bn, 87.57.uq

## I. INTRODUCTION

Coronary angiography (CA) is an interventional diagnostic procedure used to assess cardiovascular disease. During a CA procedure the primary X‐ray beam may be intercepted by the breasts depending on the tube angulation chosen. However most of the primary X‐ray beam is attenuated by tissue in the torso before reaching the breasts, thus only a small fraction of the radiation is expected to be deposited in the breasts. For certain angulations and in the breast slices further away from the chest, it is likely that scattered radiation will be the main contributor of dose to the breasts because the X‐ray beam may not intercept the breasts and because of the increased attenuation through the torso due to distance. The absorbed dose to the breast should be evenly distributed if an equal number of views are taken from the left and right side at similar angles. However, due to the slightly off‐centered position of the heart, the dose deposited in the breasts could be asymmetric.

Breast dose measurements can be found in the literature,^1^, ^2^, ^3^, ^4^ however these are normally performed using thermoluminescence dosimeter (TLD) or Monte Carlo‐based simulations with neither method expressing individual breast dose nor the breast dose distribution. Radiochromic film has the advantage of providing spatial dose information over point‐dose measurements and was used to assess breast dose distribution in coronary angiography. The effective dose from a routine CA can range from 3.4‐5.6 mSv, from previous literature.^1^, ^4^

The dose‐area product (DAP) is commonly used to monitor the output of an X‐ray tube as an indirect indicator of patient dose. It is defined as the integral dose over the area intersected by the X‐ray beam. In this instance it is used as a normalization factor for breast dose.

## II. MATERIALS AND METHODS

**Figure 1:**
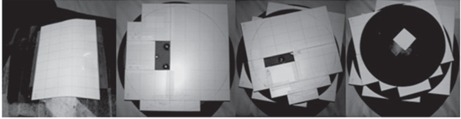
Gafchromic XR‐QA2 film placed on RANDO's chest and the subsequent breast slices. The regions not covered by Gafchromic film is the location of the pegs that hold the breast slices in place when stacked together.

**Figure 2:**
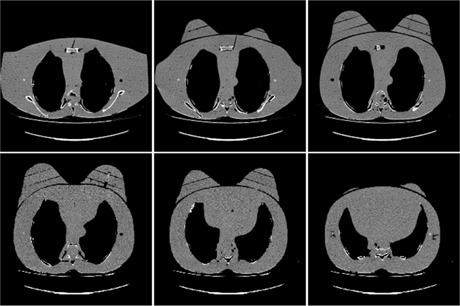
A series of CT axial slices of RANDO phantom with breast attachments in 2.5 cm increments. The breast slices sit on the chest anterior, appear slightly asymmetric, and are very rigid.

Gafchromic XR‐QA2 film (Ashland Inc., Covington, KY) was placed inside the breast slices of a RANDO phantom^(5)^ (Radiology Support Devices, Inc., Long Beach, CA) to evaluate dose distribution as shown in Fig. 1, and a series of CT axial slices of the RANDO phantom with breast attachments is shown in Fig. 2.

The film was calibrated between 0 mGy and 160 mGy on a dedicated cardiac angiography unit (Shimadzu BRANSIST Safire HF9, Shimadzu Medical Systems, Torrance, CA, with flat panel detector and filtration of 1 mm Aluminum (Rad ‐ DA protocol/BH 2 filter)) in manual mode and automatic brightness control (ABC) mode. ABC mode was selected because it is the common acquisition mode for clinical angiography units.

The film was placed side by side with a solid‐state detector (Piranha dose probe R100B, RTI Electronics AB, Mölndal, Sweden) on the patient table during the calibration. In ABC mode, beam attenuators such as PMMA, aluminum, and copper sheets were used to manipulate the system to stabilize the generator to produce the kVp most commonly seen in clinical settings.^(6)^ Solid‐state (SD) detectors tend to underestimate the dose in phantom measurement because they are less sensitive to scatter radiation from backscatter and the peripheral compared to an ionization chamber (IC).^(7)^ Correction factors to compensate for solid‐state detector response to match ionization chamber responses for the manual mode and ABC mode are 1.4 and 2, respectively. These correction factors were measured separately in a different experiment by the author^(6)^ and have been incorporated into the calibration curves. The outcome was the two calibration methods behaved in a similar manner at the dose range of interest.

**Figure 3:**
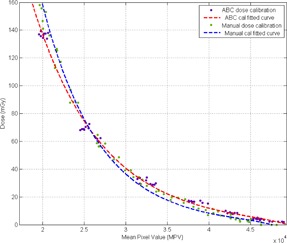
Calibration curves used to convert MPV from digitized film to dose values. The “manual cal fitted curve” was used for the controlled experiment and the “ABC cal fitted curve” was used for the mock CA procedure.

The calibrated film was scanned and digitized on an Epson V700 (Epson America Inc., Long Beach, CA) dual‐lens flatbed scanner using a reflective scanning mode in 48‐bit color, 300 dpi resolution, and no correction or color control selected.^6^, ^8^ The mean pixel values (MPV) extracted from the Gafchromic XR‐QA2 films were converted to absorbed dose using the calibration curves in Fig. 3.

A circular mask was used to ensure that only exposure within the breast slice was extracted from the film to be included in calculating the absorbed breast dose. Breast organ dose was calculated as the weighted mean dose value deposited on the XR‐QA2 film located within the breast slices. Individual breast organ doses for each left and right breast were also calculated using a mass‐weighted average method as expressed in the equations below, where $l_{1}$ stands for the mean dose measured at breast slice 1 and so on:

(1) $$D_{left}=l_{1}\times \left(\frac{0.2}{0.45}\right)+l_{2}\times \left(\frac{0.18}{0.45}\right)+l_{3}\times \left(\frac{0.07}{0.45}\right)$$

(2) $$D_{right}=l_{1}\times \left(\frac{0.25}{0.55}\right)+l_{2}\times \left(\frac{0.18}{0.55}\right)+l_{3}\times \left(\frac{0.17}{0.55}\right)$$

The breast dose was then calculated as a weighted average of the weight of each breast, $D_{\text{breast}}=(D_{\text{left}}\times 0.45)+(D_{\text{right}}\times 0.55)$. Table 1 illustrates the dimensions and weights of each breast slice.

In this experiment, tube angulations are defined into two arrangements, single and combined. A single tube angulation refers to the angle being changed in either the transverse or sagittal plane, and a combined tube angulation is where the angle changes in both transverse and sagittal planes.

A series of single and combined tube angulations based on typical tube angulations for a CA procedure^9^, ^10^ were used in the experiment and these are listed in Table 2. The fluoroscopic unit used in the experiment is the same as the unit used for the film calibrations. Manual exposures at 84 kVp, 400 mA, and 20 ms in acquisition mode at a source‐to‐image receptor distance of 100 cm and a 15 cm field of view (FOV) were used. A total of six 10‐second exposures were made per tube angulation. This was to expose the film with enough radiation to obtain a measureable dose with small uncertainty. The patient table remained in a stationary position throughout the experiment.

To assess a clinical case, a mock CA procedure was performed by an experienced cardiology registrar in ABC mode. The mock CA procedure consisted a sequence of nine acquisitions which included the seven combined tube angulations and one single angulation from the experiment, plus an additional RAO 33°/CAU 4° configuration with a 23 cm field of view to assess the left ventricle functionality (LV view) as routinely performed at the end of a CA procedure. Table 3 lists out the tube angulations and the parameters recorded during the mock CA procedure.

**Table 1:** Measurements of the breast slice attachments of RANDO

	*Left Breast*			*Right Breast*		
	*Slice 1*	*Slice 2*	*Slice 3*	*Slice 1*	*Slice 2*	*Slice 3*
Length (cm)	12.3	11	9	12.5	11	9.2
Width (cm)	11.7	10.5	8.8	11.9	10.5	9
Height (cm)	2.5	2.5	2.5	2.5	2.5	2.5
Weight (kg)	0.2	0.18	0.07	0.25	0.18	0.12
Total weight (measured)	0.45 kg (±5%)			0.55 kg (±5%)		

**Table 2:** Single and combined tube angulations used in the controlled experiment (manual mode)

LAO 10°	RAO 10°	CRA 10°	CAU 10°
LAO 30°	RAO 30°	CRA 30°	CAU 30°
LAO 40°	RAO 40°	CRA 40°	CAU 40°
PA (straight)	LAO 34°/CAU 6°	LAO 35°/CRA 31°	LAO 42°/CAU 27°
RAO 2°/CRA 30°	RAO 6°/CRA 40°	RAO 33°/CRA 4°	RAO 31°/CAU 28°

**Table 3:** Tube angulations and parameters recorded during the mock CA procedure (ABC mode) at each acquisition. Note that all acquisitions used a FOV of 15 cm except for #9, which used a FOV of 23 cm

	*Tube Angulations*	*SID (cm)*	*Tube Factors kV/mA/ms*	*No. of Frames*	*Time*	*DAP* ($\text{Gy}.\;{\text{cm}}^{2}$)
#1	LAO31°/CAU28°	101	79/ 596/ 5	35	4.7	1.22
#2	CAU39°	96	79/ 590/ 5	27	3.6	0.94
#3	RAO6°/CRA40°	102	99/ 610/ 8	34	4.5	1.19
#4	LAO35°/CRA31°	104	101/ 585/ 9	42	5.6	1.46
#5	LAO42°/CAU27°	98	87/ 628/ 5	32	4.3	1.12
#6	LAO34°/CAU6°	96	83/ 634/ 6	31	4.1	1.08
#7	RAO2°/CRA30°	99	80/ 604/ 5	39	5.2	1.36
#8	RAO33°/CRA4°	99	77/ 572/ 5	32	4.3	1.12
#9	$\text{RAO}33{}^{\circ}/{\text{CRA}4}^{C}$	99	71/ 518/ 3	131	8.7	4.57
				Total DAP		14.06

The breast dose measured for each tube angulation was then divided by the DAP reading recorded for the 60 s of exposure to calculate the normalized breast dose conversion factor for the controlled experiment. In the case of mock CA procedure, the accumulative breast dose measured for the duration of the procedure was then divided by the total DAP reading. This allows for scenarios where individual tube angulation details are not available, but a breast dose can still be estimated based on a generic routine CA procedure that uses similar tube angulations as the mock CA procedure presented here in this study.

## III. RESULTS AND DISCUSSION

**Figure 4:**
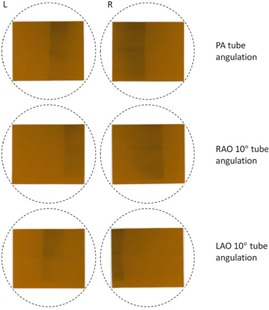
Gafchromic film placed on RANDO's chest (breast slice 1) demonstrating asymmetric breast dose as tube angulations changed from the PA position.

The Gafchromic film clearly demonstrates the asymmetric dose distribution in the sagittal and transverse planes of the breasts. Figure 4 shows the absorbed dose at the breast entrance (anterior chest) for the left and right breasts as the tube angulation changed from PA to RAO 10° to LAO 10°. This is due to the X‐ray beam exposing a larger portion of one breast as the X‐ray beam changes direction.

**Figure 5:**
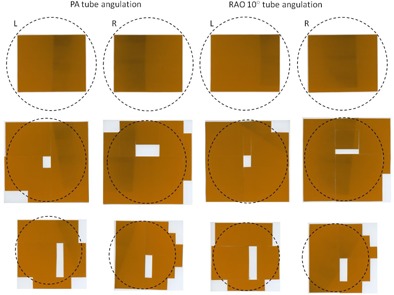
Gafchromic film placed at each of the three planes for left and right breasts for PA and RAO 10° tube angulations. The first row corresponds to breast slice 1, second row corresponds to breast slice 2, and the last row corresponds to breast slice 3. The circular mask shows the outline of the breast slice.

**Figure 6:**
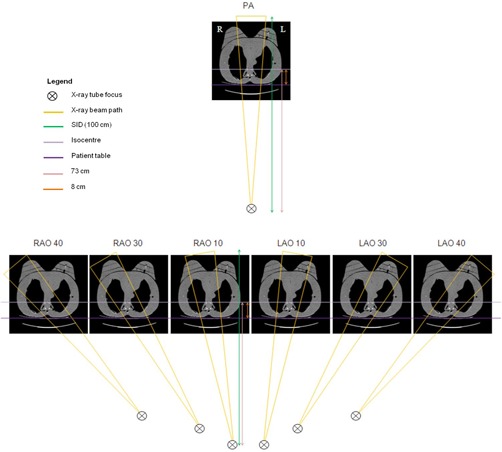
X‐ray beam path outlined for LAO‐RAO tube angulations.

Figure 5 shows the dose distribution at each of the three planes for the left and right breasts for a PA and RAO 10° tube angulation. At each sequential plane, the amount of radiation deposited in the breast is reduced due to the primary X‐ray beam being attenuated through the breast, and the X‐ray field intersecting the breast has also diminished. In the case where the tube is angled, a reduced area of the beam is intersecting one of the breasts, as demonstrated in Fig. 6.

**Figure 7:**
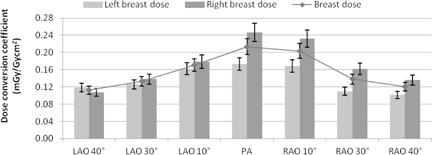
Histogram of the left, right, and overall breast dose conversions in LAO‐RAO direction. Error bars are ±8.5%.

In the experiment, the right breast measured a greater deposition of radiation dose than the left breast for almost all tube angulations investigated in this study. The mean DAP value for the single and combined tube angulations were 79.7 and $83.8\;\text{Gy}.{\text{cm}}^{2}$, respectively. Figure 7 shows the breast dose conversion factors in the LAO‐RAO direction, clearly demonstrating an asymmetric dose distribution to the breasts in the PA position where the dose to the right breast is 47% greater than the left breast. This trend continues as the tube moves towards the RAO direction but not in the LAO direction. A possible explanation for this is the left breast is slightly smaller in dimension compared to the right breast, thus a fraction of the X‐ray beam is deposited outside the breast boundary and does not contribute to the left breast dose. Considering the right breast is almost completely out of the primary X‐ray beam in the LAO direction for large angles, it is assumed that the dose measured in the right breast under these circumstances arises from internal scattering by the tissues inside the phantom. This would also be true in the RAO angulations in regards to the scattered radiation deposited in the left breast.

One anomaly is LAO 40°, where the left breast measured a slightly higher dose than the right. This could be a statistical error, as both measurements overlap their respective uncertainty range. The overall breast dose appears to behave more quadratic in the LAO direction than in the RAO direction.

**Figure 8:**
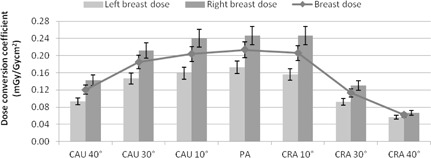
Histogram of the left, right, and overall breast dose conversions in the CAU‐CRA direction. Error bars are ±8.5%.

Figure 8 is the breast dose conversion factors in the CAU‐CRA direction and it illustrates a difference in dose between the left and right breasts that remains quite consistent, between 40% and 56% greater in the right breast than the left, with the exception of CRA 40°, where the difference is only 16%. Since the X‐ray tube is not biased to either side of the patient where one breast can be exposed to more primary beam radiation than the other in the LAO‐RAO configurations, this confirms the asymmetric distribution of radiation to the breasts. The overall breast dose in the CRA direction drops off much quicker than the CAU direction, which drops off in a quadratic manner. This is because in the CRA position, the X‐ray beam is greatly attenuated by tissue in the abdomen before reaching the breasts, whereas in the CAU position, the X‐ray beam is mainly traversing the lungs, which have less attenuating property compared to the abdomen region.

**Figure 9:**
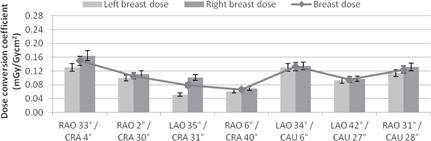
Histogram of left, right, and overall breast dose conversions for the combined tube angulations. Error bars are ±8.5%.

Figure 9 reveals that the relationship with breast dose for combined tube angulations is not as simple to discern. As the cranial angle increases, the overall breast dose decreases, irrespective of the LAO‐RAO component, suggesting a strong influence in attenuation behavior in the cranial angle when combined tube angulations are involved. For combined tube angulations with a CAU component, the same influence is not apparent. Instead a decreasing angle in LAO component seems to dominate the breast dose behavior, suggesting a stronger influence by the LAO‐RAO angulations when caudal angles are involved in a combined effect. However due to the limited projections investigated in combination with CAU, the findings here are not definitive.

**Figure 10:**
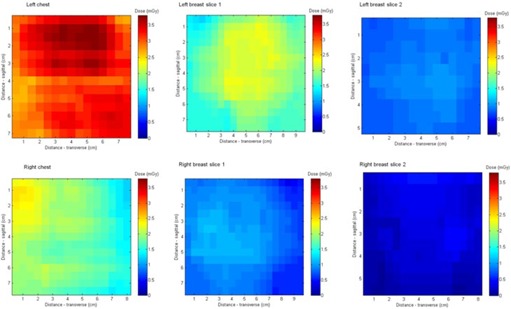
Dose distribution of the left and right breasts in three planes from the mock CA procedure. The left breast received 2.3±6.5% mGy, the right breast received 1.17 mGy±6.5%, and the overall breast dose was 1.7±6.5%.

Contrary to the experiment, the left breast had a higher dose than the right breast in the mock CA procedure, as shown in Fig. 10, and also demonstrates hot spots within the breast planes. The total DAP for the mock CA procedure was $14.06\;\text{Gy}.{\text{cm}}^{2}$ and the measured left, right and overall breast doses were 2.3, 1.17, and 1.7 mGy, respectively. These measurements resulted in a normalized dose conversion factor of $0.16\;\text{mGy}/\text{Gy}.{\text{cm}}^{2}$ for the left breast, $0.08\;\text{mGy}/\text{Gy}.{\text{cm}}^{2}$ for the right breast, and a normalized overall breast dose of $0.12\;\text{mGy}/\text{Gy}.{\text{cm}}^{2}$. Breast dose measurements from the literature^1^, ^2^, ^3^, ^4^ ranged from 0.03 to $0.12\;\text{mGy}/\text{Gy}.{\text{cm}}^{2}$. The findings from this study are in line with the values found in the literature, although the approach of using Gafchromic film to measure breast dose in coronary angiography hasn't been used before.

Applying the breast dose conversion factors from the controlled experiment to the DAP values recorded for the mock CA procedure (column 7, Table 3), the breast dose was calculated as 1.7±0.15 mGy. For acquisition #9, the breast dose conversion factor for the 15 cm FOV was used, since there were no data for the larger FOV. While the actual measurement made from the Gafchromic film in the mock CA procedure resulted in the same breast dose (1.7±0.11 mGy).

The DAP value is a measure of the X‐ray tube output and is dependent on various factors such as geometry, X‐ray field area, X‐ray generator parameters, patient thickness, table attenuation, ABC feedback, exposure time, and complexity of the interventional case. These factors will affect the patient dose and, in turn, the breast dose received by the patient. Since the breast dose will be proportional to the DAP value and it is common to use normalized DAP for dose conversion factors, thus the proposed breast dose conversion factors in this paper are normalized to DAP. Realistically, the breasts are not rigid and do not sit upright like the RANDO phantom when lying supine, but rather fall to the side of the patient. Therefore the breast dose conversion factors could be higher than the expected values quoted in this study.

DAP‐to‐effective dose conversion factors can often be found in the literature. As an example, a typical DAP value for a CA is $82\;\text{Gy}.{\text{cm}}^{2}$ at the hospital where this study was performed. If a DAP‐to‐effective dose conversion factor of $0.2\;\text{mSv}/\text{Gy}.{\text{cm}}^{2}$ ^(4)^ and the breast dose conversion factor of $0.12\;\text{mGy}/\text{Gy}.{\text{cm}}^{2}$ are applied, the resultant effective dose would be 16.4 mSv and the breast dose 9.8 mGy.

The main difference between the controlled experiment and the mock CA procedure is the use of the floating patient table top. During a clinical procedure, the floating table top is enabled to allow easy manipulation of patient movement. Therefore the cardiologist may position the patient in such a manner that the X‐ray beam spends more time on the left or right side of the patient. However in the controlled experiment, the floating patient table top was locked in a fixed position to allow reproducibility of phantom positioning. This explains why the right breast measured a higher dose in the controlled experiment, but a higher dose in the left breast was measured in the mock CA procedure. This suggests the contribution to the asymmetric distribution of the breast dose is not solely dependent on the tube angulation used, but also dependent on the table translation movements which can result in one breast always receiving a greater radiation dose than the other at the discretion of the operator.

An advantage of using Gafchromic film is the ability to capture two‐dimensional dose distribution, whereas TLDs can only provide a single dose point measurement unless a large quantity of TLDs are placed inside the organ of interest. Also, the primary beam and scattered beam radiation deposited in the breast is more readily depicted by Gafchromic film and can be quantified. Monte Carlo methods are a convenient tool for calculating absorbed organ dose and effective dose without the need to make physical measurements if the necessary parameters are made available (e.g., patient size, tube geometry, distances, X‐ray field size, X‐ray output, etc.). But unlike Gafchromic film, most Monte‐Carlo‐based simulations only provide a single absorbed breast dose value; thus asymmetry of dose distribution will not be revealed. Thus Gafchromic film can be used both qualitatively and quantitatively.

Limitations of the Gafchromic film are that it is nonreusable, unlike TLDs or Monte Carlo simulations, and the minimal dose required for the film to be readable within an uncertainty of 15% was found to be at least 10 mGy in this experiment.

## IV. CONCLUSIONS

Asymmetric breast dose distribution was successfully demonstrated using Gafchromic XR‐QA2 film during a mock coronary angiogram procedure and for a collection of single and combined tube angulations frequently used in CA. The controlled experiment provided a method for estimating the left and right breast dose, as well as the overall breast dose, if the DAP and tube angulation are known per acquisition.

If DAP and tube angulation are unknown, the application of a typical CA would give a normalized dose conversion factor of $0.16\;\text{mGy}/\text{Gy}.{\text{cm}}^{2}$ for the left breast and $0.08\;\text{mGy}/\text{Gy}.{\text{cm}}^{2}$ for the right breast. Thus the overall breast dose conversion factor is $0.12\;\text{mGy}/\text{Gy}.{\text{cm}}^{2}$.

Asymmetric breast dose distribution is dependent on tube angulation and is related to the use of the floating patient table top by the operator.

## COPYRIGHT

This work is licensed under a Creative Commons Attribution 4.0 International License.
